# Meeting of the Fulton County Medical Society

**Published:** 1915-02

**Authors:** R. R. Daly


### FULTON COUNTY MEDICAL SOCIETY.

Regular Meeting February 4, 1915.

By R. R. Daly, M. D.

Dr. E. G. Jones spoke on "Cysts on the Mesentery" and illustrated three cases that he reported upon. Skiagrams and drawings were shown, indicating the conditions and eliminating the diagnosis in each case.

Dr. W. P. Nicolson said that he had never, so far as he knew, seen a mesenteric cyst. He may have passed them without recognition, but that is doubtful. Jones is to be congratulated upon having seen so many. It is fortunate that they remain well after operation.

Dr. A. H. Bunce said that the cysts are difficult to pass upon after operation as to their structure. If the contents have been present for any length of time, the cell walls are likely to have changed and the distinguishing linings to have disappeared. They may follow accidents and be really haematomata. Chylous cysts are dilatations of villi, but the lacteal character of the walls would soon disappear under pressure. The cysts come from different sources and assume similar characteristics because of the similar nutrition and position they occupy. He quotes Dowd as saying they are hydatid, embryonic and cystic malignant. The one he saw could not be classed as dermoid, though that sort of cyst might be present in the mesentery.

Dr. W. S. Goldsmith said that his experience was like Nicolson's and he had never seen a mesenteric cyst.

Dr. E. C. Thrash said that debris and ameba can get into chyle ducts and make retention cysts that occupy the mesenteric position. He saw one removed by Dr. Kime some time ago, which was of that character. Traumatism may cause them, but haematomata are likely to be absorbed unless infected.

Dr. E. L. Griffin said that he had a child with a growth in the abdomen causing occlusion of the bowel. He thought it a sacromatous kidney at first, but found it a mesenteric cyst pressing on the gut. It could not be ennucleated and he had to resect the gut. The cyst was filled with clear yellowish fluid. The child recovered and later took a prize at a children's show.

Dr. G. M. Niles exhibited several skiagrams showing "Some Interesting Gastro-Intestinal Conditions."

Dr. L. Amster said that each picture was worthy of a paper in itself, and that Niles is to be congratulated upon pushing this kind of work. The different shapes of the stomach were interesting. The long tube-like stomach causes pain and the cases are often asthenic. The pains are located and the pictures taken, but the lesions are often in remote places. Sometimes they are not even in the stomach. Pictures of cancer tell the story only when the disease is far advanced and there is little to be done. This is due largely to the fact that such cases do not talk of their trouble early enough and at this time the internists have not been making X-Ray examinations of slight gastric complaints as a routine matter. Several pictures should be taken and thus an early diagnosis may be made that would save life by indicating operation early enough to be of avail. Appendicitis is difficult to determine by the X-Ray. There is not enough to cause a shadow.

Dr. H. R. Donaldson said that ulcers of the stomach are not readily determined by X-Ray and that over 80 per cent of the Mayo's cases were proven clinically before operation. It would be of greatest assistance to have them located in advance by the skiagram because they are often difficult to see when the stomach is opened. He observes that there is a fine advance along this line of research and he expects soon to see gastric surgery upon a scientific basis.

Dr. H. I. Battey said he had thought the X-Ray was infallible when positive, but recently he had seen a skiagram of the splenic flexure attached to the diaphragm. They moved up and down together before the fluoroscope. Operation showed a band attached to the spleen. The X-Ray and its manipulators should have more time. As to the short circuiting operations on the gut the X-Ray has sometimes indicated. He had seen several of those in Patterson's clinic in London where the remaining colon was not in good condition. He thought the complete removal better in the first instance.

Dr. W. P. Nicolson said he had had some experiences with the X-Ray that were far from being satisfactory and that he was not enthusiastic about it. It had its uses and doubtless its future would be great, but at present it could not decide as to an appendicitis and it was likely to be misleading in other abdominal conditions. He told of two instance where there seemed to be vesceral displacement, but operation showed parts in place. He has opened the abdomen at least three thousand times and has learned to rely upon clinical signs and indications. He hopes the X-Ray in the future will be in closer accord with these.

Dr. E. C. Davis said that one of Niles' pictures confirmed exactly the operative work in an old man where exploration would have been fatal. With the information given, the work was done successfully.

Dr. Niles closed saying that he had shown three cases of appendicitis that proved correct, but as Nicolson said it can not always be done. As to fallacious conclusions, people must be careful in making them. Great clinical experience in addition to the X-Ray pictures should avoid mistakes. The detail of the pictures themselves should be well understood by the examiner and that takes time to learn and to become accustomed to. Surgeons should remember that surgery was being done before the building of the pyramids, while X-Ray is still an infant science. It is a good child, however, and Niles is doing the best he can with it. He said that Thomas was the greatest of the Disciples because he really wanted to know and put his hands to things. If we all do that, we shall learn more of the X-Ray's powers. There are more things in heaven and earth than any of us have dreamed of and it is only by keeping busy with everything we can learn that we can keep in touch with scientific advances.

REGULAR MEETING FEBRUARY 18, 1915.

The Society had as its guest Dr. Hugh H. Young of Baltimore. Dr. Young devoted himself to an "Illustrated Discussion of the Surgery of the Prostate."

This was complete and exhaustive and comprised the regional anatomy of the parts and the fundamentals of the various operations that one is called upon to do in that neighborhood. It is needless to say that the Perineal Method was the only one advocated from Dr. Young's standpoint. He is too well known in his wide teaching to discuss that point. He did show exactly why he believed that avenue the best and safest by which to reach the prostate and bladder and the most commodious for work. He pointed out its dangers as well and agreed with his critics that it required a constant familiarity with the work to be successful.

He was never arbitrary in his statements and even said for the abdominal surgeon who was familiar only with the anterior wall in his regular work, the suprapubic method was the best. He even showed how much might be done in that way, but at the same time indicated what might be overlooked or not so well done. His description of his hospital and method of work and study was inspiring to his audience and his entire talk was received with the utmost appreciation.

The discussion later was participated in by Drs. Nicolson, McRae, Huguley, Ballenger and Block and many interesting questions were asked and answered informally.

Later in the evening the entire Society was at the Capital City Club, where a buffet supper was served and where everyone had opportunity to meet and converse with Dr. Young. And let it be said that the pleasure Dr. Young gave in these cordial personal meetings complemented the enthusiasm his scientific address had aroused.

---

### THE PRIZE PREVARICATOR.

It is often said that no phase of human nature will ever surprised the experienced physician, but it is probable that the judge of the divorce court is, if possible, more immune against shock.

More ingenuity seems to enter into the testimony in the divorce trial than was ever exercised by Baron Munchausen or any other writer of fiction.

It is the pleasant duty of the divorce court to sift out the few grains of real fact from the mass of conflicting testimony and exercise judgment surpassing that of Solomon in making a decision.

It is quite likely that the elaboration which is shown in framing up plausible court testimony is responsible for many a miscarriage of justice where the real, uncolored facts are disclosed. The habit of discount becomes so fixed that the truth is not recognized by the Court and the honest client suffers for the sins of others.—Monthly Cyclopedia.

